# Validation of a Perception of Objectification in the Workplace Short Scale (POWS)

**DOI:** 10.3389/fpsyg.2021.651071

**Published:** 2021-06-07

**Authors:** Lola Crone, Lionel Brunel, Laurent Auzoult

**Affiliations:** ^1^Laboratory Epsylon EA 4556, Department of Psychology, University Montpellier, Montpellier, France; ^2^Laboratory Psy-DREPI EA 7458, Department of Psychology, University Bourgogne, Dijon, France

**Keywords:** objectification in the workplace, questionnaire, action, social perception, dehumanization

## Abstract

An increasing number of studies focus on the phenomenon of objectification in the workplace. This phenomenon reflects a process of subjection of the employee, where he is considered as an object, a mean (utilitarian) or reduced to one of his attributes. Previous studies have shown that objectification can have consequences on the workplace health or performance. Field studies are based on objectification measures based on tools whose psychometric qualities are unclear. Based on a previous workplace objectification measurement scale, we conducted a study with the aim of devising a new parsimonious scale. We present three studies which aim to validate this new scale. In the first study, an EFA and a CFA were performed in order to construct a scale and verify its structure validity. We obtained a 10-item scale reporting two factors labeled “Instrumental value” and “Powerfulness.” The psychometric qualities of this scale were satisfactory, i.e., showed good internal reliability and good structural validity. In a second study, we tested the convergent and divergent validity of the scale. We observe that POWS is adequately correlated with dehumanization indicators. Finally, in a third study, we found that only powerfulness was associated with negative consequences for occupational health. This suggests that objectification is a process of social perception that contributes either to the devaluation of social agents in the workplace or to normal functioning at work.

## Introduction

Objectification is a form of dehumanization (Haslam, 2006; Volpato and Andrighetto, 2015) which is expressed through a relationship of subjugation or a reductive perception of a person based on one of their attributes (Nussbaum, 1995). It occurs when the person is viewed as an object. In this context, over and above the denial of their humanity, i.e., passivity, denial of subjectivity and denial of autonomy, the person is viewed through their use, i.e., instrumentalization, possession or interchangeability, or their form, i.e., reduction to appearance, body or silence (Langton, 2011). In the workplace, objectification consists in behaving with an employee as if the latter had no thoughts or emotions, as if they had to be controlled in order to act, deprived of initiative, exploitable and malleable at will. Regarding reduction to form, persons are viewed solely through their appearance or their body, and are not listened to, as they are judged incapable of expressing their feelings about their work or themselves.

We find similar patterns of behavior evoking sexual objectification. At the same time, all these behaviors have in common to rely on elementary perceptual processes (Bernard et al., 2018). We present a series of studies which aim to clarify the measurement of objectification in workplace. We started from a scale measuring the usual behaviors of objectification in workplace (Auzoult and Personnaz, 2016a) and we developed a new scale based on the perception of employees in workplace.

### Origins of Objectification

Several explanatory hypotheses have been put forward to account for objectification. Objectification is thought to be a means of reducing complexity, of coping with uncertainty, and consequently of facilitating interaction by enabling others to be perceived *via* simple attributes. Landau et al. (2012) highlight the fact that managers, who anticipate difficulties in carrying out their duties and in considering the subjectivity of their employees, focus on their professional attributes (skills for example). A similar explanation can be found in the medical field where objectification is described as a defense mechanism which is established when faced with the difficulty of delivering care effectively, for example when care involves hurting the patient (Timmermans and Almeling, 2009; Haque and Waytz, 2012).

Other authors consider that objectification is associated with the exercise of power. Gruenfeld et al. (2008) observed through several experiments that the exercise of power leads to the objectification of others, that’s to say perceiving them through the sole dimension of their use in achieving goals which are set by the person who holds the power. Other field studies (Auzoult and Personnaz, 2016a) have highlighted the fact that working in an organization based on strict respect for authority, rationality of procedures, division of work and written formal communication is associated with self-objectification. Moreover, the link between power and objectification is conditioned by the interpersonal attitude of leaders (Sanders et al., 2015) and more generally by the quality of interpersonal relationships (Renger et al., 2016). Beyond power, we can emphasize that scientific discourse on management is underpinned by a vision of the employee as a resource for the organization and not as an end in themselves (Cheney and Carroll, 1997; Shields and Grant, 2010; Rochford et al., 2016).

Following on from the analyses of the thinker Marx (1944), the sociologist Durkheim (1893) and the psychoanalyst Fromm (1956), objectification can be considered as a corollary of the organization of work activity. Haque and Waytz (2012) situate the origin of medical dehumanization at the level of care activity. This highlights the fact that medical care promotes deindividuation (anonymity of the uniform, no name), the perception of the patient as diminished (illness), dissimilarity (caring/neat status, sick/healthy) or labeling (denomination by the disease). Other authors emphasize the impact of labor robotization (Moore and Robinson, 2016) or the characteristics of employees, such as their age (Wiener et al., 2014). Several experimental studies (Andrighetto et al., 2017, 2018; Baldissarri et al., 2017b) have highlighted the fact that objectification takes place when observing someone working in an activity which is continually reproduced (repetitiveness), which is divided into several basic units carried out separately (fragmentation) and whose pace of execution and planning depends on another person or a technical system rather than on the employee themselves (external control). This process occurs if the activity takes place in an industrial context (machine based) rather than a craft-based context (the manufacture of a specific object) and does not exclusively concern the perception of others. So Baldissarri et al. (2017a) highlighted the fact that being placed in a situation of performing an activity which is repetitive, fragmented or under the control of someone else leads to a perception of oneself as self-objectified/dementalized, like an instrument (a tool, a thing, or a machine), and as having little personal freedom, another attribute specifically associated with human beings. The latter element is important as this study by Baldissarri and al. also underlines the fact that seeing oneself as generally having little personal freedom reinforces the phenomenon of self-objectification. There is therefore a downward spiral in which the feeling of loss of freedom may lead to perceiving oneself as an object, this state in turn reducing the feeling of freedom, *etc*.

### Consequences of Objectification

When considering the consequences of objectification in the field of work, it can be seen that this type of relationship is associated with “cognitive deconstructive” states (Christoff, 2014), with a loss of perceived humanity (Loughnan et al., 2017), occupational burnout (Baldissarri et al., 2014; Szymanski and Mikorski, 2016; Caesens et al., 2017), decrease of job satisfaction and depression (Szymanski and Feltman, 2015), sexual harassment (Wiener et al., 2013; Gervais et al., 2016), and self-objectification (Auzoult and Personnaz, 2016a). Self-objectification constitutes dementalization, i.e., a feeling of having lost the capacity to act, to plan, to exercise control over oneself or one’s environment, or to feel emotions (Gray et al., 2011).

Some studies have also considered the positive consequences, such as personal empowerment (Inesi et al., 2014) or employability (Rollero and Tartaglia, 2013; Nistor and Stanciu, 2017). In the medical field, objectification is also conceived as a means of facilitating medical care (Haque and Waytz, 2012). In these cases, objectification is considered to be a provider of psychosocial resources or to facilitate activity at work.

### Objective of the First Study

There is therefore still much research to be done to understand both the antecedents and outcomes of objectification. This work requires the use of valid and simple tools to study the phenomenon of objectification at work.

**FIGURE 1:**
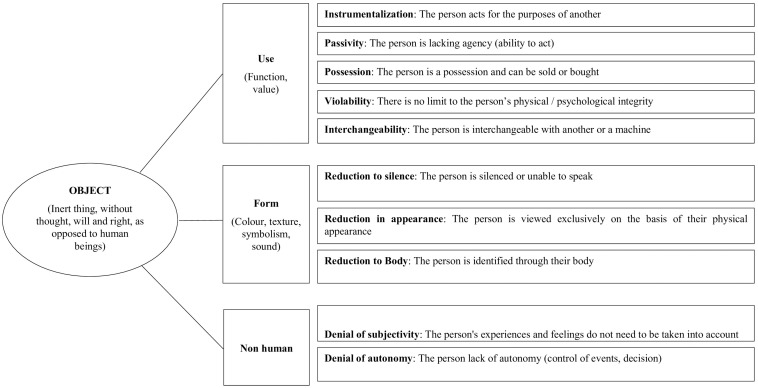
Theoretical dimensions of objectification.

In this study, we aim to construct a scale of objectification in the workplace. We have taken as a basis the perception of being objectified scale created by Auzoult and Personnaz (2016a) which adopts and extends the adaptation of the scale of Gruenfeld et al. (2008) and Baldissarri et al. (2014). In the original scale, we only took into account the instrumentalization that is thought to be the core defining component of objectification. In relation to the work of Nussbaum (1995) and Langton (2011), Auzoult and Personnaz have developed a scale that also takes into account the processes associated to objectification: denial of agentivity, usefulness or his/her apparent characteristics (Figure 1 and Table 1). According to this scale, objectification at work means that the employee acts according to the objectives of a third party and is considered incapable of taking initiatives or decisions. In the same way his/her affects, his/her health and his/her ideas are disregarded and he/she is considered solely from the point of view of his/her appearance. Finally, he/she would be considered as interchangeable with others or with a machine, because he/she belongs to his/her organization. In this way of thinking, the employee is a resource just like raw material or capital (Shields and Grant, 2010; Moore and Robinson, 2016).

**TABLE 1:** List of Auzoult and Personnar scale items with their associated dimensions: Instumentalization, passivity, possession, violability, interchangeability, reduction to silence, reduction in appearance, reduction to body, denial of subjectivity, and denial of autonomy.

**Property of the object**	**Dimensions of objectification**	***No.***	**Items**
Form	Reduction to appearance	1	At work my boss and/or my colleagues only consider me on the basis of my physical appearance
Use	Instrumentalization	2	My boss and my colleagues appreciate me even when I’m not useful to him/her (R)
Form	Reduction to silence	3	My boss and my colleagues never ask my opinion at work, as though I had nothing to say
Non-human	Denial of autonomy	4	My boss and my colleagues never ask if I would like to work in a different way
Use	Possession	5	I sometimes have the impression that I am the possession of my employer and that I will easily be transferred or sold to another company
Use	Interchangeability	6*	At work, my boss and/or my colleagues give me the impression that my work could be replaced by that of a machine
Non-human	Denial of subjectivity	7	At work, my boss and my colleagues act as if my private life was of no importance and shouldn’t be taken into account
Form	Reduction to silence	8	My boss and my colleagues do not listen to what I have to say about my work
Use	Instrumentalization	9*	My boss and/or my colleagues think more about what I can do for them than what they can do for me
Non-human	Denial of autonomy	10	My boss and my colleagues tell me how to do my work even when I do not ask anything
Form	Reduction to body	11*	For my boss and/or my colleagues, what I feel or what I think is of little importance. What counts is that I am physically able to work
Use	Interchangeability	12*	In my workplace, my boss and/or/my colleagues think that if I was replaced by a machine, the work would be done just as well, or even better
Use	Instrumentalization	13	The relationship with my boss and/or my colleagues is based on the fact that we appreciate one another from a human point of view rather than on the fact that I am productive (R)
Form	Reduction to body	14*	My boss and/or my colleagues consider that my physical aptitudes are my only skills
Non-human	Denial of autonomy	15	My boss and my colleagues give me no latitude in my work as they think that I would not know how to do it differently
Non-human	Denial of subjectivity	16	My boss and/or my colleagues are often interested in what I feel because they want to get as close to me as possible (R)
Form	Reduction to appearance	17	The only thing that counts in my workplace is that I present myself well physically
Use	Violability	18*	At work, my boss and/or my colleagues act as if my health was of no importance and should not be protected
Use	Instrumentalization	19*	If I was no longer useful to my boss and/or my colleagues, my relationship with them would come to an end
Non-human	Denial of subjectivity	20	At work, people make me do as they wish without asking me if I want to or I like doing it
Use	Passivity	21	At work, my boss and/or my colleagues reflect back the image of someone who is subject to events and incapable of taking the initiative
Use	Instrumentalization	22	My boss and/or my colleagues consider the relationship they have with me to be important because it allow them to achieve their objectives
Use	Violability	23*	My health and my physical sate are of secondary importance for my boss and/or my colleagues
Use	Possession	24*	It’s as if my employment contract made me into an object or a product which my employer could dispose of as they see fit
Use	Passivity	25	At work, my boss and/or my colleagues, behave with me as someone to whom one says what must be done and who always follows suit
Use	Instrumentalization	26*	My box and/or my colleagues only seek me out when they need something

*Items retained in the short version of the scale.

This scale has been used in several studies (Auzoult and Personnaz, 2016a,b; Auzoult, 2019). It seems to benefit from good internal consistency (Cronbach α from 0.90 to 0.91). However, the factor structure that is presented in the original study reveals the existence of five factors while theoretically 10 dimensions were expected (e.g., instrumentalization, passivity, etc., see Figure 1 and Table 1). The authors (Auzoult and Personnaz, 2016a) considered that the correlations observed between the items justified the existence of a single factor of objectification at work. This leads us to consider the scale as relatively complex. Similarly, the scale contains 26 items which can penalize field research aimed at populations uncomfortable with writing. These different findings led us to withdraw the 26 items of the original scale and to collect a sufficient number of answers to carry out a new validation study of the scale.

## Study 1: Objectification Short Form Scale Construction

The first study consisted in developing an abbreviated version of the objectification perception scale. First, we evaluated the psychometric properties of the original perception of objectification scale. The scale includes 26 items (Table 1) which measure the 10 theoretical dimensions of objectification: instrumentalization, reduction in appearance, denial of autonomy, denial of subjectivity, passivity, interchangeability, violability, possession, and reduction to body and reduction to silence. Respondents used seven-point scales ranging from “not at all” (1) to “quite” (7). Secondly, if the confirmatory analysis of the previous scale did not fit the data, an exploratory analysis was conducted to refine this. A new confirmatory analysis on a replication sample was performed to test the new version.

## Materials and Methods

### Statistical Method

EFA and CFA were carried out using the R software by the following packages: Psych (Revelle, 2015), Lavaan (Rosseel, 2012). The parallel analysis allowed us to determine the number of factors to be extracted. We have selected factors with an eigenvalue greater than 1, according to Kaiser’s criteria. The items were deleted if their unique variance was <0.60, their saturation coefficient >0.60 and their double load <0.10 on a second factor. THE EFA extraction method was maximum likelihood and oblimin rotation. For confirmatory analysis, our models were estimated using the following statistical indices: the chi-square and degree of freedom (CMIN/DF), the comparative fit index (CFI), the tucker-lewis index (TLI), and the root mean square error of approximation (RMSEA). A good model with a CMIN/DF < 3, has a CFI, GFI and TLI value >0.90 and RMSEA values below 0.08. To evaluate the reliability of the scale, we used cronbach’s alpha which must be >0.7

### Participants and Procedure

780 participants (385 males and 395 females) participated in the study. Their average age was 38. Regarding their level of education, 48.9% of them had a level of education lower than the bachelor’s degree, 22.6% a bachelor’s degree, and 28.4% a level higher than the bachelor’s degree. Regarding their status, 72.4% of them were non-managerial, 19.3% were executive and 8.2% were senior managers. 84.9% worked in a service activity and 21.5% in industry. The work objective scale was sent *via* mailing lists to employees working in various sectors of activity. Respondents were contacted via the researchers’ networks and then asked to extend the study via their own networks (snowball technique).

The questionnaire allowed us to measure the study variable and participants’ characteristics. The answers were anonymous. Respondents received a report on the study’s main results by email. Four participants that had not fully completed the questionnaire, were withdrawn from the study.

## Results

### Confirmatory Factor Analyses (CFA) of the Perception Objectification Scale in the Workplace

The Table 2 summarizes the CFA indices from the scale of the perception of objectification in the workplace (Auzoult and Personnaz, 2016a) to a one-factor. The CMIN/DF must be less than 3, but the index indicates that it is 6.43. The RMSEA is 0.083, it can be considered satisfactory, as it must be less than or equal to 0.08. Nevertheless, the CFI is less than 0.09 which is not satisfactory. The 1-dimensional objectification perception scale can be considered inadequate with regard to the indicators presented.

**TABLE 2:** Fit indices for de confirmatory analyses of the Perception of objectification on the workplace (Auzoult and Personnaz, 2016a) (*N* = 780).

**Model one factor**	**X^2^ (p)**	**df**	**x/df**	**CFI**	**RMSEA**
	1922.29	299.00	6,43	0.788	0.083

### Development and Validation of a Short-Scale Perception of Objectification in the Workplace

#### Sample

To refine the scale and improve the exploratory analysis, we split the sample in two. The participants were randomly numbered so that the first sample contains even numbers and the second contains odd numbers. The first sample (*n* = 390) will be used for the exploratory analysis, the second sample (*n* = 390) will be used for the confirmatory analysis.

#### Exploratory Factor Analyses on POWS (Perception Objectification in Work Scale)

**FIGURE 2:**
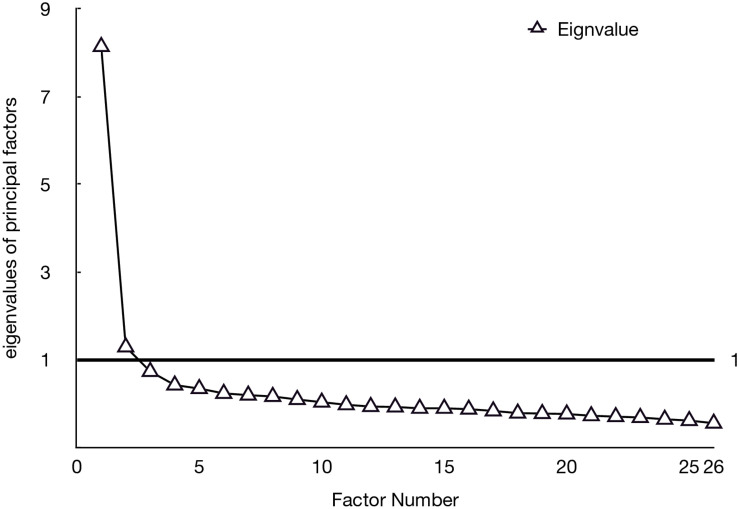
Distribution of eigenvalues relative to factors.

The factor extraction analysis proposed a two-factor model (Figure 2). The first factor explains 23% of the variance and has an eigenvalue of 6.06. The factor 2 has an eigenvalue of 3.53 and explains 14% of the variance. The Table 3 shows the factors loading of the 10 items selected from the initial scale.

**TABLE 3:** Factor loadings estimates for POWS with oblimin rotation (*N* = 390).

POWS*/10 items/2factors	**Instrumental value**	**Powerfullness**
Item 23	**0.72**	0.02
Item 19	**0.71**	–0.09
Item 26	**0.70**	–0.05
Item 18	**0.70**	0.02
Item 14	**0.70**	–0.06
Item 24	**0.70**	–0.05
Item9	**0.67**	–0.02
Item 11	–0.01	**0.71**
Item 12	–0.10	**0.73**
Item 6	0.07	**0.60**
Loading/Eigen Values	6.06	3.53
Cumulative variance	0.23	0.37

*POWS, Perception of Objectification at Work Short scale.

The first factor consisted of 7 items (26, 24, 23, 19, 18, 14, and 9). These represented instrumentalization (26, 19, and 9), possession (24), violability (23 and 18), and reduction to body (14). The saturation coefficients of this factor were between 0.67 and 0.72. The second factor consisted of 3 items (12, 11, and 6). The items represented interchangeability (6 and 12), and reduction to body (11). The saturation coefficients for this factor were between 0.60 and 0.73. The first factor included items that referred to the utility and/or importance of the employee for others: we labeled this factor ‘‘Instrumental value^1^.’’ The second factor grouped items that referred to the power of the employee, whether physically assessed or compared to that of machines: we labeled this factor ‘‘Powerfulness^2^.” For a 2-factor model, the KMO index is 0.90, Bartlett’s test is less than 0.05 which can be considered excellent.

#### Confirmatory Analysis

**FIGURE 3:**
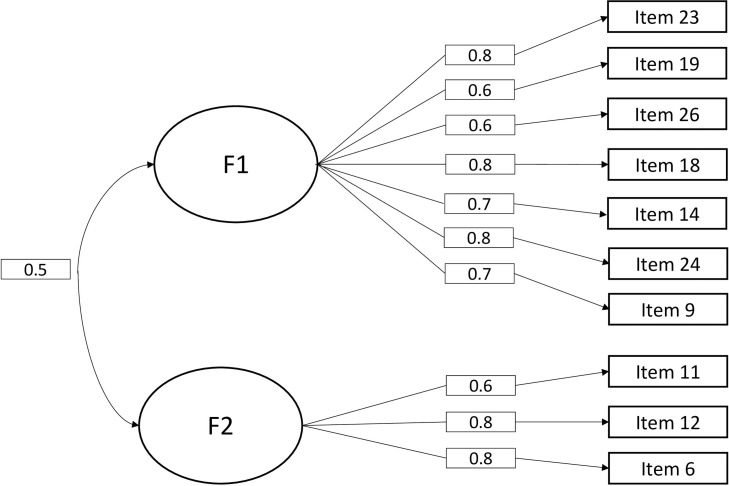
Structural model of the objectification scale after confirmatory analysis.

The confirmatory analysis was performed on the second sample to analyze the fit of the 2-factor model in 10 items. The indices of the 2-factor model were compared to a 1 factor model. The Table 4 shows that the 2-factor model requires better adjustment indices. Referring to the threshold of the recommended values, the analysis showed that the CMIN/DF was satisfactory because it was less than 3 (χ^2^ = 77.599, DF = 34, CMIN/DF = 2.28). The CFI was also satisfactory because it was higher than 0.9 (CFI = 0.973) as was the GFI (GFI = 0.963). RMSEA was of good quality because it was less than 0.08 (RMSEA = 0.041). Finally, the RMR was less than 1 (RMR = 0.041). Compared to 1-factor model, the 2-factor model indicates a better adequacy of the indices. Then, the internal reliability of our model (Figure 3) showed us that the correlations of the intra-factor items were all greater than 0.60 with their latent factor. The inter-factor correlation was equal to 0.50. Finally, the internal consistency index of the various factors, expressed by the Cronbach’s alpha coefficient, showed us that factor 1 had an alpha of 0.80 and factor 2 had an alpha of 0.70.

**TABLE 4:** Fit indices for the confirmatory factor models of the POWS questionnaire (N = 390).

**Models tested**	**X^2^ (P)**	**df**	**x/df**	**CFI**	**GFI**	**TLI**	**AIC**	**RMSEA**
2 *–* factor	132.30	34	3,8	0.967	0.968	0.956	26325.601	0.061
1 *–* factor	498.17	35	14,23	0.844	0.882	0.800	26689.475	0.130

#### Comparisons of Mean POWS Score for Age, Sex, Professional Status, and Level of Study

Comparison of means across subjects (Table 5) shows that enforcement agents and managers have a higher score of perceived objectification at workplace than upper managers (execution agent: *t* = 2.95, *df* = 82.50, *p* < 0.005; manager*: t* = 2.07, *df* = 132.18, *p* < 0.05). For gender, males have a significantly higher score than females (*t* = 2.68, *df* = 757.26, *p* < 0.05). Regarding age, we do not observe any difference in POWS scores between the different age groups, however the under 25s have a higher score of the powerfulness factor than the 41/50s (*t* = 1.91, *df* = 430.16, *p* < 0.05). Finally, concerning the level of study, we only observe a difference in average between bachelor’s degree levels and postgraduate education, the first having a higher average in the powerfulness factor (*t* = 2.51, *df* = 288.3, *p* < 0.05).

**TABLE 5:** Mean (standard deviation) for POWS score depending on age, sex, working status, and degree of study (*N* = 780).

	***N***	**POWS**	**F1: Instrumentality**	**F2: Powerfulness**
**Age**				
<25	221	2.50 (1.08)	2.85 (1.28)	1.68(0.97)*
26–40	174	2.61 (1.10)	3.01 (1.37)	1.67 (1.01)
41–50	230	2.59 (1.12)	3.05 (1.41)	1.52 (0.83)
>51	155	2.57 (1.07)	2.99 (1.31)	1.58 (0.92)
**Working status**				
Execution agent	562	2.61(1.09)**	3.01(1.34)*	1.67(0.97)***
Manager	150	2.54(1.09)*	2.99 (1.36)	1.49 (0.90)
Upper manager	64	2.23 (0.97)	2.62 (1.30)	1.31 (0.60)
**Sex**				
Female	395	2.46 (1.01)	2.88 (1.28)	1.50 (1.78)
Male	385	2.67(1.67)*	3.08(1.42)*	1.72(1.06)**
**Bachelor’s degree**				
<Bachelor	220	2.63 (1.06)	3.05 (1.33)	1.63 (0.89)
Bachelor	174	2.58 (1.13)	2.93 (1.36)	1.72(1.06)*
>Bachelor	380	2.52 (1.09)	2.95 (1.35)	1.52 (0.89)

*p < 0.05, **p < 0.01, and ***p < 0.001. POWS, Perception of Objectification at Work Short scale.

## Discussion

The objective of this first study was to construct a parsimonious objectification at the workplace level with an unambiguous factor structure. We took as a basis for our work the scale constructed by Auzoult and Personnaz (2016a), which initially included 26 items measuring an alleged general objectification factor.

First, we conducted a confirmatory analysis of the model to ensure its psychometric properties. The indices showed us that the 1-factor model was not adequate. An exploratory and confirmatory analysis led us to choose a 10-item two-factor structure. This two-factor structure does not contradict the idea that objectification is a unitary phenomenon since these two factors are strongly intercorrelated. As regards criteria validity, the mean comparison of our sample shows us elements in line with the literature. Indeed, we observe that the executing agents have a higher mean of objectification than the upper managers. This result supports the very definition of objectification, which stems from a fragmented, repetitive activity but also from a power relationship in which subordinates are treated exclusively on their usefulness in achieving a goal (Magee and Galinsky, 2008).

The structural validity of this new scale is satisfactory. The following two studies aim to establish the convergent and divergent validity of the scale by taking into account other psychological constructs supposed to be associated with objectification at work.

## Study 2: Convergent and Divergent Validity

To verify the convergent and divergent validity of our scale, we have compared the POWS scale to the following elements: the dehumanization and humanization scales. We postulated that the correlations would be significantly positive between the POWS scale and the dehumanization scale (convergent validity) and the correlations would be significantly negative between the POWS scale and the humanization (divergence validity). We expect these relationships which have already been observed by Auzoult (2019). Indeed, the fact of perceiving oneself as objectified by one’s professional entourage is positively associated with a perception of oneself as an instrument (i.e., mechanical dehumanization) and negatively associated with the perception of oneself as a person (i.e., humanization).

## Materials and Methods

### Participants and Procedure

74 participants participated in our study (12 men and 61 women). The average age is 37. As regards professional status, we have 23% of executive agents, 43% of middle managers, 11% of upper managers, 12% of artisans and 11% of students. We distributed our questionnaire on social networks, specifying that the answers were anonymous. The contact procedure was similar to the previous study.

### Measures

#### Perception of Objectification

The perception of objectification was measured with the previously validated 10-item POWS scale with an internal consistency index of 0.92.

#### Mechanical Dehumanization and Humanization

A 2-dimensional scale of instrumentality and humanness that measures the perception of being seen as an instrument (mechanical dehumanization) or a human being (humanization) (Andrighetto et al., 2017). Five words are presented to describe oneself as a human person (human being, individual, and person) and five words to describe oneself as an instrument (tool, thing, machine). The first factor (mechanical dehumanization) has an internal consistency index of 0.90 and the second factor (humanization) has an internal consistency index of 0.77. Participants respond using a likert scale ranging from 1 «not at all” to 7 “quite.”

## Results

The Table 6 shows the correlations between objectification (POWS scale), the 2 subjacent dimensions, mechanical dehumanization and humanization. Objectification (POWS scale) is positively associated to mechanical dehumanization (*r* = 0.75) and negatively associated to the humanization (*r* = −0.55). The two sub−dimensions of the POWS scale are also positively associated to the mechanical dehumanization (Instrumentality: *r* = 0.73; Powerfulness: *r* = 0.63) and negatively associated to humanness (Instrumentality: *r* = −0.54; Powerfulness: *r* = −0.44). These results confirm a satisfactory convergent and divergent validity of our scale.

**TABLE 6:** Correlations between perception of objectification at work (POWS scale), dehumanization, and humanization (*N* = 74).

	**POWS**	**Factor 1: Instrumentalization**	**Factor 2: Powerfulness**
Dehumanization	0.75***	0.73***	0.63***
Humanness	−0.55***	−0.54***	−0.44***

***p < 0.001. POWS, Perception of Objectification at Work Short scale.

## Discussion

The objective of this second study was to establish the convergent and divergent validity of our new scale. These are satisfactory, however, in view of our sample (*N* = 74), comparisons in other larger samples with the same measures of similar constructs will be included in further studies.

Concerning the two subdimension of the POWS scale, we have labeled these two factors “Instrumental value” and “Powerfulness.” The first factor refers to a feeling of being reduced to a mere support or physical object (like a tool) and thus being represented in a work environment more as tool than a social agent. Thus, the agent feels as if he/she is being utilized and valuated in his/her working environment. The second factor refers to the feeling of not being recognized as an agent in the working environment. Thus, the agent thinks that recognition comes solely from their actions. This leads us to two observations. On the one hand, objectification refers to a representation of people which reflects the process of dehumanization where the employee perceives himself/herself as a resource at the service of others or to a process of comparative devaluation of the employee in his or her technical universe. These two representations of objectification are not strictly similar. Indeed, the fact of perceiving oneself as a resource at the service of the organization can be considered relatively usual since the employment contract reflects this organizational objective. If employees adhere to organizational goals, this phenomenon of reducing the person to a means may even be considered functional and acceptable (Orehek and Weaverling, 2017). In some cases, it can even be conceived that objectification can lead to an increase in the market value of the employee (Rollero and Tartaglia, 2013) or an increase in the feeling of self-efficacy (Nistor and Stanciu, 2017). On the contrary, perceiving oneself as having less power than a machine represents an attack on the instrumental value of the worker. From this point of view, the consequences of objectification for health are not necessarily unambiguous depending on whether we consider the two factors that make up our scale.

This observation led us to question one of the observations made about the consequences of the objective for health. Specifically, one of the consequences of objectification is self-objectification. Self-objectification is thought to take place through dementalization, which is to say, a perception of the self as being incapable of feeling or thinking about work. In studies in which objectification and mentalization are measured, there is a relatively weak relationship (<0.30) (Baldissarri et al., 2014; Auzoult and Personnaz, 2016b) or an absence of a relationship between these two psychological constructs (Auzoult, 2019). We think that this difficulty in observing a strong and consistent relationship is due to the fact that the different dimensions of objectification, “Instrumental value,” and “Powerfulness” are not equivalent from the point of view of the consequences for health, here dementalization at work. Specifically, we think that only the “Powerfulness” dimension of objectification is systematically negatively associated at the level of mentalization, which results in self-objectification at work. The aim of this third study was to test this hypothesis using the reduced scale (POWS) and self-objectification at work.

## Study 3: Relationship Between Objectification and Self-Objectification at Work

## Materials and Methods

### Participants and Procedure

A total of 650 participants (307 females, 343 males) participated in the study. They were on average 38 years old. Regarding education level, 31% had a level of education lower than the bachelor’s degree, 43% a bachelor’s degree and 25% a level higher than the bachelor’s degree. Regarding status, 69% of them were executive, 21% were middle managers and 9% were senior managers.

Participants were invited to complete an online questionnaire. The questionnaire allowed us to measure the study variables. The answers were anonymous and once data were completed and results processed, respondents received a report of the study’s main results by email.

### Measures

#### Perception of Objectification at Work

Objectification was measured using the short scale POWS developed in the previous study. It consisted of 10 items and participants responded using 7-point scales ranging from “not at all” (1) to “quite” (7). This scale is composed of 2 factors: “Instrumental value” with an internal consistency index of 0.87 and “Powerfulness,” with an internal consistency index of 0.73.

#### Self-Objectification

Self-objectification was measured using the Self-Mental State Attribution Task (Baldissarri et al., 2014). The scale is based on 19 items, with an internal consistency index of 0.89, allowing the attribution of different mental states during a working day (for example: “To what extent have you been likely to feel psychological states during a working day?: *feel a need*; *have an intention; reasoning*”). Participants responded using seven-point scales ranging from “not at all” (1) to “quite” (7).

## Results

The two indicators of objectification were positively associated (*r* = 0.40). Self-objectification was significantly negatively associated with powerfulness (*r* = −0.11) and tendentially with instrumental value (*r* = −0.07) (Table 7). We conducted a regression analysis considering self-objectification as a dependent variable and the two dimensions of objectification as independent variables. Powerfulness (β = −0.09) explained self-objectification (Table 8). Our hypothesis was confirmed.

**TABLE 7:** Correlations between variables: self-objectification, instrumental value, and powerfulness (*N* = 650).

	***M***	***SD***	**α**	**1**	**2**	**3**
1. Self-obj certification	4.58	0.87	0.89	**–**	−0.07*	−0.11**
2. Instrumental value	2.99	1.34	0.86			0.40**
3. Powerfulness	1.59	0.91	0.73			**–**

*p < 0.10 and **p < 0.05.

**TABLE 8:** Regression analysis – self-objectification as dependent variable (*N* = 650).

	**B**	***SE B***	**P**
*Constant*	4.79	*0*.*09*	
Instrumental value	–0.02	*0*.*03*	–0.03
Powerfulness	–0.09	*0*.*04*	−0.09*
Model R^2^		0.012*	

*p < 0.05.

## Discussion

In this third study the objectification was measured using the reduced scale. We measured dementalization at work as a consequence of objectification. It was observed, as expected, that only the second factor named powerfulness was systematically associated with self-objectification (dementalization), that is to say with negative consequences for occupational health. This result confirms that it is not the fact of perceiving oneself as a means of serving others that is problematic for occupational health. It appears that it is the fact of perceiving oneself as comparable to an instrument or a machine that is deleterious to health at work. From this point of view, self-objectification appears as the result of a comparative devaluation of social agents in their technical universe. However, a single study is not a sufficient basis to draw a conclusion, further studies are needed to confirm this first result.

## General Discussion

The two factors involved in the measurement of objectification refer to the value of the employee in reference to the action he/she can perform for others, but also to his/her lack of value that reflects the lack of importance of his/her health or replaceability by machines. Work on social perception leads to the idea that common sense knowledge is evaluative and utilitarian in the sense that it does not aim for accuracy but for action (Funder, 1987). The process of objectification thus appears as the product of an action-oriented evaluative activity, that is to say endowed with functionality as has been previously emphasized (Haque and Waytz, 2012). According to “economy of action” (e.g., Proffitt, 2006), individuals perceive their environment through its opportunity for action and its associated cost. In this process, individuals integrate each source (i.e., physical or social) reducing the cost of their action depending on the nature of the task (e.g., Meagher and Marsh, 2014; Osiurak et al., 2014). This is probably why objectification occurs in contexts where control of action is salient (power, relational uncertainty) and where production through activity is based on the coordination of the human and the technical.

The objectification process refers to a perceptive activity in which the person is encoded through his/her form (structural dimension) but also and above all through his/her functional properties (semantic dimension) that is to say in accordance with the same processes as the perceptive activity of objects (Humphreys et al., 1997; Martin, 2007). Numerous studies have suggested that people think about objects in much the same way as they think about people (Lakey and Orehek, 2011; Orehek and Forest, 2016). When action is concerned, people are used in the same way as objects and are evaluated according to their instrumental usefulness for achieving others’ goals (Orehek and Weaverling, 2017). Objectification in the workplace therefore involves a process of social perception that accounts for basic perceptual mechanisms. We thus find a frame of reference similar to the process of sexual objectification (Bernard et al., 2018). From this point of view, the scale that we have developed takes a closer look at the phenomenon of objectification at work, which can then be considered as a process of social perception in the work context. This also leads to the dissociation of the objectification process from its immediate consequences, i.e., instrumentalization, violability, interchangeability, denial of agency, and so on.

## Conclusion

The purpose of this study was to construct a short scale for objectification in the workplace. At the end of this validation study we obtained a scale of 10 items with a bifactorial structure whose psychometric qualities are very satisfactory. From the point of view of content, this scale seems to comprehend the process of objectification as a process of social perception. In future studies, therefore, it would be better to dissociate the objectification process from its consequences in terms of social behavior at work. Likewise, the fact that objectification accounts for the value of people in their work context leads to an interesting question that we need to address experimentally: is objectification a loss of social value or an absence of social attribution?

## Data Availability Statement

The raw data supporting the conclusions of this article will be made available by the authors, without undue reservation.

## Ethics Statement

Ethical review and approval was not required for the study on human participants in accordance with the local legislation and institutional requirements. The patients/participants provided their written informed consent to participate in this study.

## Author Contributions

LC, LB, and LA participated in the design of the study and analyzed the results and wrote the manuscript. LC conducted the experiment. All authors read and approved the final manuscript.

## Conflict of Interest

The authors declare that the research was conducted in the absence of any commercial or financial relationships that could be construed as a potential conflict of interest.

## Footnotes

1 Item 26 “My boss and/or my colleagues only seek me out when they need something”; item 24 “It’s as if my employment contract made me into an object or a product which my employer could dispose of as they see fit”; item 19 “If I was no longer useful to my boss and/or my colleagues, my relationship with them would come to an end”; item 14” For my boss and/or my colleagues, what I feel or what I think is of little importance, what counts is that I am physically able to work.”; item 9 “ My boss and/or my colleagues think more about what I can do for them than what they can do for me”; item 23 “ My health and my physical state are of secondary importance to my boss and/or my colleagues. “; item 18 “ At work, my boss and/or my colleagues act as if my health was of no importance and should not be protected.”

2 Item 11 “ My boss and/or my colleagues consider that my physical aptitudes are my only skills”; item 12 “ In my workplace, my boss and/or my colleagues think that if I was replaced by a machine, the work would be done just as well, or even better”; item 6 “ At work, my boss and/or my colleagues, give me the impression that my work could be replaced by that of a machine.”
